# Agreement of Ultra-Short-Term Heart Rate Variability Recordings During Overseas Training Camps in Under-20 National Futsal Players

**DOI:** 10.3389/fpsyg.2021.621399

**Published:** 2021-02-05

**Authors:** Yung-Sheng Chen, Jeffrey C. Pagaduan, Pedro Bezerra, Zachary J. Crowley-McHattan, Cheng-Deng Kuo, Filipe Manuel Clemente

**Affiliations:** ^1^Department of Exercise and Health Sciences, University of Taipei, Taipei, Taiwan; ^2^Exercise and Health Promotion Association, New Taipei City, Taiwan; ^3^College of Health and Medicine, School of Health Sciences, University of Tasmania, Hobart, TAS, Australia; ^4^Escola Superior Desporto e Lazer, Instituto Politécnico de Viana do Castelo, Viana do Castelo, Portugal; ^5^The Research Centre in Sports Sciences, Health Sciences and Human Development, Vila Real, Portugal; ^6^School of Health and Human Sciences, Southern Cross University, Lismore, NSW, Australia; ^7^Department of Medical Research, Taipei Veterans General Hospital, Taipei, Taiwan; ^8^Department of Medicine, Taian Hospital, Taipei, Taiwan; ^9^Tanyu Research Laboratory, Taipei, Taiwan; ^10^Instituto de Telecomunicações, Delegação da Covilhã, Lisboa, Portugal

**Keywords:** heart rate variability, autonomic nervous system, overseas training camps, futsal training, ultra-short-term recording

## Abstract

**Background:** Monitoring the daily change in resting heart rate variability (HRV) can provide information regarding training adaptation and recovery status of the autonomic nervous system (ANS) during training camps. However, it remains unclear whether postural stabilization is essential for valid and reliable ultra-short-term (HRV_UST_) recordings in short-term overseas training camps.

**Design:** Observational and longitudinal study.

**Purpose:** This study aimed to investigate ultra-short-term heart rate variability recordings under stabilization or post-stabilization periods in four overseas training camps.

**Participant:** Twenty-seven U-20 male national team futsal players voluntarily participated in this study.

**Method:** Resting HRV was evaluated for 10 min during the early morning of each training camp. The natural logarithm of the root mean square of successive normal-to-normal interval differences (LnRMSSD) was used for comparisons. Time segments of HRV were divided into two periods with three measures within each: (1) the first 30-s (1st_30 s LnRMSSD), the first 60-s (1st_60 s LnRMSSD), and the 5-min standard (1st_5 min LnRMSSD) during stabilization; (2) the first 30-s (2nd_30 s LnRMSSD), the first 60-s (2nd_60 s LnRMSSD), and the 5-min standard (2nd_5 min LnRMSSD) after stabilization.

**Result:** The results demonstrated trivial to small ES (−0.03; 0.46), very large to nearly perfect ICC (0.76; 0.98), and narrow range of SEM (0.06; 0.31) when all time segments of HRV_UST_ were compared to the 1st_5 min and 2nd_5 min HRV. Furthermore, the magnitude of the correlation coefficients ranged from very high to nearly perfect for all the time segments (*r* = 0.83; 0.97). The HRV_UST_ posted excellent agreement in all time segments (bias = −0.05; 0.12) with/without postural stabilization. Trivial to small levels of effect size in all time segments of LnRMSSD_mean_ (0.02; 0.41 ES) and LnRMSSD_cv_ (−0.49; −0.02 ES) across overseas training camps was identified.

**Conclusion:** The first 30 or 60-s LnRMSSD recordings can be used to evaluate daily cardiac-autonomic function during overseas training camps in futsal players. The process for stabilization seems to be unnecessary for measuring the morning resting LnRMSSD in overseas training camps among young adult futsal players.

## Introduction

Heart rate (HR) variability (HRV) is a neurophysiological marker that reflects the cardiac-related activation regulated by the autonomic nervous system (ANS) (Buchheit, 2014). Assessment of HRV requires recording a time series of HR beat-to-beat intervals (R wave-to-R wave interval, RRI) via a non-invasive electrocardiographic device (ECG) or a HR monitor sensor. The measurement of HRV can help sports practitioners and coaches interpret autonomic function in terms of training adaptations (i.e. increase in RRI is associated with improvement of aerobic capacity) (Sandercock et al., 2005; Plews et al., 2013a), recovery status (Buchheit et al., 2007; Nakamura et al., 2009; Chen et al., 2019), and autonomic health during international competitions (Flatt and Howells, 2019; Flatt et al., 2019; Muñoz-López et al., in press).

The standard process to collect HRV data requires a 5-min short-term recording period preceded by a 5-min stabilization period (Task Force of the European Society of Cardiology the North American Society of Pacing Electrophysiology., 1996). However, this assessment method is limited to specific circumstances, i.e., during traditional training schedules, due to the time-consuming nature of this type of assessment. Yet, with recent methodological developments, the assessment of ultra-short-term HRV (HRV_UST_), HRV recording of <60 s, have been established to improve the usability of this technique in field-based settings (Castaldo et al., 2019).

In a practical sense, HRV_UST_ assessment during sports training can be used to understand cardiac modulation during exercise or physiological adaption after an acute and chronic training regime. Nakamura et al. (2015) reported excellent limits of agreement and acceptance in 1-min HRV_UST_(natural logarithm of the root mean square of successive normal-to-normal interval differences, LnRMSSD_UST_), compared to a criterion of 5-min LnRMSSD (a 5-min HRV record after 5-min stabilization). The correlation between changes in LnRMSSD_UST_ (i.e., 0–1; 1–2; 2–3; 3–4; 4–5 min) and LnRMSSD criterion was between 0.45 and 0.75, with the highest value (*r* = 0.75; 90% CI: 0.55–0.85) found between LnRMSSD_UST_ at 1–2 min and LnRMSSD criterion, indicating measurement validity and agreement of HRV_UST_ assessment after 1-min of stabilization. In addition, Krejčí et al. (2018) compared LnRMSSD_UST_ every 30 s in 5-min stabilization records to a 5-min criterion (6–10 min) in 30 endurance athletes at the national level (10 ski runners, 8 road cyclists, and 12 cross-country skiers) and 30 university students. When LnRMSSD_UST_ and HR were measured in the supine position, the minimal stabilization period to stabilize both indices was 60-s for endurance athletes and 90-s for university students for valid and reliable estimations. Interestingly, Pereira et al. (2016) demonstrated excellent reliability and limits of agreement of 1-min HRV_UST_ during 5-min stabilization in 35 elite futsal players. An additional observation in an athletic population reported by Flatt and Esco (2016) also supports this notion. However, interpretation of these findings may be limited due to the heterogeneity of the sample pools from mixed genders and training regimens. Despite the time efficiency of data collection using HRV_UST_, there are no standard recommendations for using HRV_UST_ with or without stabilization.

Monitoring the daily change in resting HRV can provide information regarding training adaptation and recovery status of ANS health during national team training camps (Flatt and Howells, 2019; Flatt et al., 2019). During a national team training camp, soccer players who played more than 60 min of match play experienced augmented ANS modulation (by measuring daily morning LnRMSSD) after a friendly match. This profound effect lasting for 72 h after (Muñoz-López et al., in press). Thus, it is important to monitor the HRV parameters for assessing recovery status during training camps. It was recently observed that 30-s mean value of LnRMSSD (LnRMSSD_mean_) measure was accurate and valid compared to the standard 5-min LnRMSSD measure during both training camps (domestic and overseas) and international competition in a male U-20 national futsal team. In contrast, coefficient of variation of LnRMSSD (LnRMSSD_cv_) measure required at least 2 min recordings to provide valid and reliable HRV measures during training camps (Chen et al., 2020; Clemente F. M et al., 2020). However, the interpretation of these findings were highlighted as being after 5-min postural stabilization period.

Monitoring LnRMSSD_mean_ and LnRMSSD_cv_ can help us to understand the vagal-related adaptation and psychophysiological status associated with physical fitness during training period (i.e., increase in LnRMSSD_mean_, decrease in LnRMSSD_cv_, and improvement of aerobic capacity) (Nakamura et al., 2020). Most of previous studies reported agreement of HRV_UST_ based on crossectional designs, in this study, weekly HRV_UST_ of LnRMSSD_mean_ and LnRMSSD_cv_ was investigated during four overseas training camp in a male U-20 national futsal team. In light of the abovementioned studies, the purpose of this study was twofold. Firstly, to compare HRV_UST_ recordings within stabilization and after stabilization periods during short-term overseas training camps in U-20 national futsal players as a surrogate to the traditional 5-min standard measures. Secondly, to compare the variation of HRV_UST_ measures among short-term overseas training camps. It was hypothesized that HRV_UST_ recordings during stabilization and after stabilization would show similar degrees of agreement and reproducibility to 5-min HRV records. The secondary hypothesis was that HRV_UST_ would show similar characteristics among overseas training camps.

## Materials and Methods

### Experimental Approach to the Problem

This study was a cross-sectional and observational study. Morning resting HRV was measured in four overseas training camps prior to the Asian U-20 Futsal Championship final. The HRV_UST_ assessment during the first 5-min (stabilization) and second 5-min (after stabilization) was compared. The time segments of HRV records were divided into the first 30-s (1st_30 s LnRMSSD), the first 60-s (1st_60 s LnRMSSD), and the 5-min recording (1st_5 min LnRMSSD) during stabilization, and the first 30-s (2nd_30 s LnRMSSD), the first 60-s (2nd_60 s LnRMSSD), and the 5-min recording (2nd_5 min LnRMSSD) after stabilization.

The number of participating players varied from camp to camp due to budgeting and logistical issues (1st training camp: 6 days, 15 players; 2nd training camp: 5 days, 20 players; 3rd training camp: 6 days, 17 players; 4th training camp: 10 days, 14 players). The players involved in the fourth oversea training camp were the final registered players for the continental tournament. All assessments were conducted during the overseas training camps prior to the 2019 Asian Football Confederation U-20 Futsal Championship Final (Tabriz, Iran). Table 1 shows detailed information regarding the schedule of four overseas training camps.

**Table 1:** The schedule of overseas training camps prior to the continental tournament final.

**Location**	**Date**	**Duration (days)**	**Players (numbers)**	**Training sessions (sessions)**	**Friendly matches (games)**
1st TC Shenzhen, China	July 28th–August 2nd 2018	6	15	1 session (115-min)	4 games (89, 100, 101, 91-min)
2nd TC Nagoya, Japan	November 19th−23th 2018	5	20	3 sessions (109, 128, 126, 76-min,)	3 games (97, 75, 77-min)
3rd TC Osaka, Japan	April 7th−12th 2019	6	17	4 sessions (91, 108, 95, 117-min)	4 games (107, 101, 102, 90-min)
4th TC Luso, Portugal	June 1st−10th 2019	10	14	3 sessions (83, 73, 90-min)	5 games (87, 82, 78, 83, 67-min)

*TC, training camp*.

### Experimental Procedure

During each oversea training camp, the players were lodged in a domestic hotel one night before international travel. All players undertook daily resting HRV measures before breakfast in the early morning during the training camps. The players were instructed to maintain a comfortable sitting position for resting HRV assessment. The players were informed to control their breathing with their preferred patterns while their eyes were closed. The morning resting HRV was recorded via an individual portable Polar HR monitor (Polar team Pro, Polar Electro, Kemple, Finland). All sensors were synced to a Polar team pro dock. All data were uploaded to a secure cloud server and then subsequently exported to a laptop for data analysis. The duration of the resting HRV assessment was 10-min. All measures were performed in a quiet and spacious meeting room between 7 a.m. and 8 a.m. in local time.

### Participants

Twenty-four outfield players and three goalkeepers, male futsal players were recruited and voluntarily participated this study from Chinese Taipei U-20 national futsal team (mean ± standard deviation: age = 17.93 ± 0.87 yrs; height = 1.71 ± 0.07 m; body weight = 65.39 ± 9.39 kg; body fat = 12.54 ± 2.76 %; maximal aerobic capacity = 51.98 ± 3.07 ml^.^kg^−1.^min^−1^). The players signed informed consent forms and were all familiarized with experimental procedures prior to participation. The study was approved by the Human Ethics Committee of the University of Taipei (UT-IRB-2018-068) and undertaken in accordance with the Declaration of Helsinki and its later amendments in 2013.

### Heart Rate Variability

A portable telemetric HR monitor system was used to record the resting HRV (Polar team Pro, Polar Electro, Kemple, Finland). Each player was issued an HR sensor and HR strap for the entire duration of the training camp. Kubios HRV analysis software (Premium version 3.2.0., Kubios, Kuopio, Finland) was used to calculate LnRMSSD. Artifact correction was set at a medium threshold level, and the window width was set at 300 s with a window overlap of 50%. Smoothing priors set at 500 Lambda were used for detrending methods (Tarvainen et al., 2014). If the percentage of ectopic beats in daily measure were >5%, then the data was excluded from the analysis.

### Statistical Analyses

Descriptive data of the measured variables are presented as LnRMSSD_mean_ or LnRMSSD_cv_ and standard deviation (SD). Inter-differences of HRV_UST_ to standard values was analyzed by using Cohen's d effect size (ES). The level of ES was interpreted as trivial (0.0–0.2), small (0.2–0.6), moderate (0.6–1.2), large (1.2–2.0), very large (>2.0) (Hopkins et al., 2009). For reliability analysis, interclass correlation coefficients (ICC) with a two-way random model and a single measure were used to determine relative reliability. The level of ICC values were assessed as nearly perfect (0.9–1), very large (0.70–89), large (0.50–69), moderate (0.31–49), and small (0–0.3) (Hopkins et al., 2009). Moreover, the standard error of measurement (SEM) was used to analyzed the absolute values of reliability. The SEM was calculated as ${\text{SD}}^{.}\sqrt{1-\text{ICC}}$ (Weir, 2005). For validity analysis, the relationship of HRV recordings between each time segment were assessed via Pearson product-moment correlation coefficient (*r*). The magnitude of the correlation coefficients was determined as trivial (*r* < 0.1), small (0.1 < *r* < 0.3), moderate (0.3 < *r* < 0.5), high (0.5 < *r* < 0.7), very high (0.7 < *r* < 0.9), nearly perfect (*r* > 0.9), and perfect (*r* = 1) (Hopkins et al., 2009). In addition, Bland–Altman plots were used to evaluate the upper and lower limits of agreements among all time segments of LnRMSSD (Bland and Altman, 1986). Statistical analyses were conducted using by SPSS® Statistics version 25.0 (IBM, Armonk, NY, USA) and Microsoft Excel 2016 (Microsoft Corporation, Redmond, WA, USA).

## Result

### Agreement and Reliability of Ultra-Short-Term Heart Rate Variability

**Figure 1:**
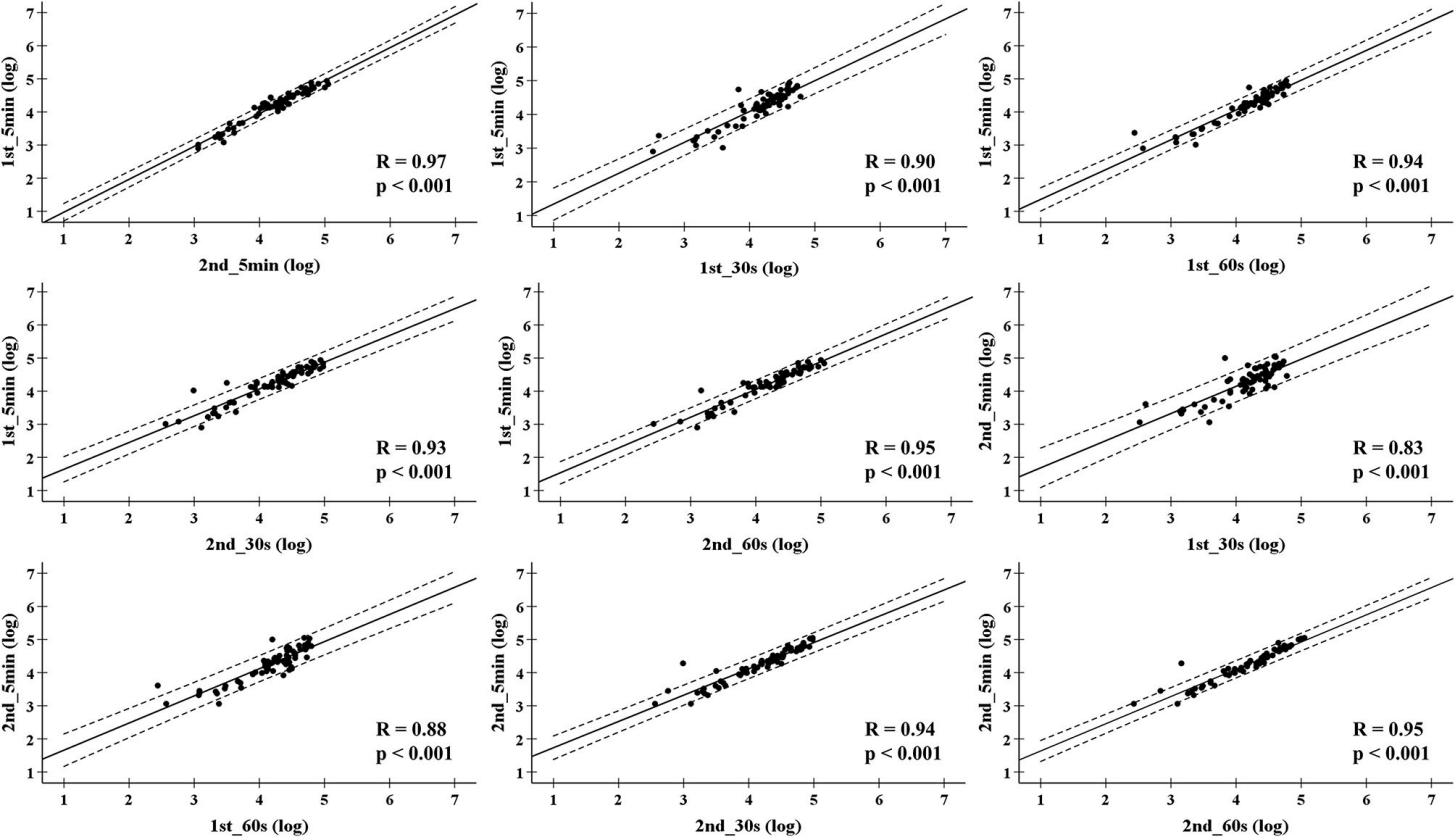
Pearson correlation coefficient for the mean of natural logarithm of the root mean square differences between adjacent normal R-R intervals in all time segments during four overseas training camps. 1st_5 min = The first 5-min LnRMSSD; 1st_30 s = The first 30 s LnRMSSD; 1st_60 s = The first 60 s LnRMSSD; 2nd_5 min = The 6-10 min LnRMSSD; 2nd_30 s = The secondary 30 s LnRMSSD; 2nd_60 s = The secondary 60 s LnRMSSD.

**Figure 2:**
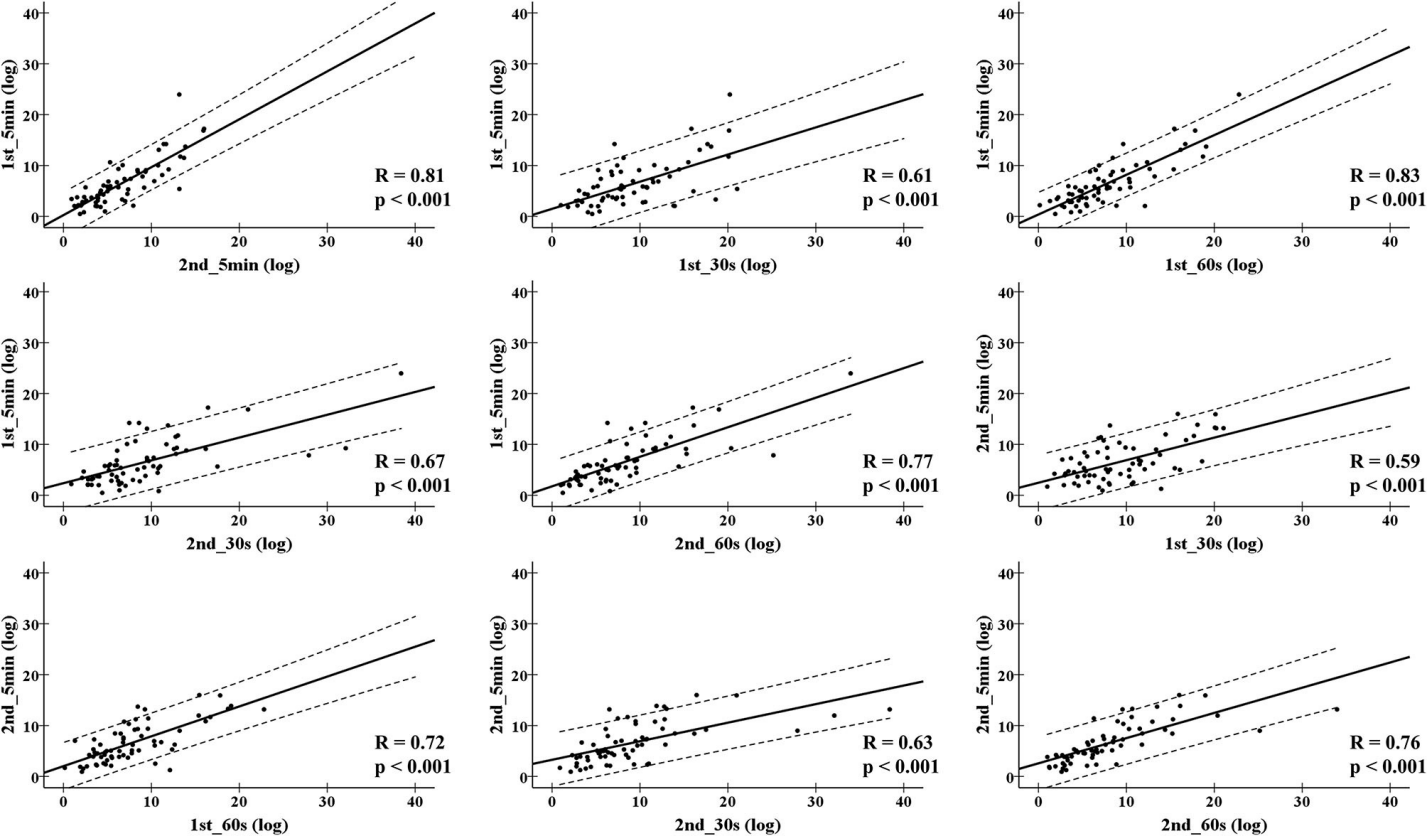
Pearson correlation coefficient for the coefficient of variation of natural logarithm of the root mean square differences between adjacent normal R-R intervals in all time segments during four overseas training camps. 1st_5 min = The first 5-min LnRMSSD; 1st_30 s = The first 30 s LnRMSSD; 1st_60 s = The first 60 s LnRMSSD; 2nd_5 min = The 6-10 min LnRMSSD; 2nd_30 s = The secondary 30 s LnRMSSD; 2nd_60 s = The secondary 60 s LnRMSSD.

The daily HRV recordings from all players were compliance with full attendance during the training camps. For LnRMSSD_mean_, small ES and very large ICC values were found between the 1st_30 s and 1st_5 min, or 1st_30 s, and 2nd_5 min in the 2nd training camp. A similar finding was reported in the 3rd training camp, except for the ICC comparison of the 1st_30 s and 1st_5 min. Moderate ES and very large ICC values were also found between the 2nd_30 s and 1st_5 min, or the 1st_30 s and 2nd_5 min. The other comparisons showed trivial ES and nearly perfect ICC values (Table 2). For LnRMSSD_cv_, a wide range of ES (−0.91; −0.02) and ICC (0.23; 0.88) values were found among the overseas training camps (Table 3). In Figure 1, the results of LnRMSSD_mean_ exhibited nearly perfect correlations in all correlations analyses except for a very high correlation between the 2nd_5 min and 1st_30 s (0.83, *p* < 0.001), and 2nd_5 min and 1st_60 s (0.88, *p* < 0.001). In Figure 2, the results of LnRMSSD_cv_ demonstrate high (0.59–0.77, *p* < 0.001) to very high (0.72–0.83, *p* < 0.001) correlations in all comparisons.

**Table 2:** Mean of natural logarithm of root mean square differences between adjacent normal R–R intervals (LnRMSSD) during 0–30 s, 0–60 s, and 0–5 min criterion in stabilization (1st_5 min) and after stabilization (2nd_5 min) assessments.

**Training camp**	**Parameters**	**ES (90% CI)**		**ICC (90% CI)**		**SEM**		Bias (± 1.96_*_SD)	
		**1st_5 min**	**2nd_5 min**	**1st_5 min**	**2nd_5 min**	**1st_5 min**	**2nd_5 min**	**1st_5 min**	**2nd_5 min**
1st training camp (*n* = 15)	1st_30 s	−0.13 (−0.73; 0.47)*	−0.08 (−0.69; 0.52)*	0.87 (0.93; 0.99)^‡^	0.82 (0.61; 0.92)^‡^	0.14	0.17	−0.06 (−0.52; 0.40)	−0.05 (−0.61; 0.52)
	1st_60 s	−0.14 (−0.74; 0.46)*	−0.10 (−0.70; 0.50)*	0.95 (0.88; 0.98)^§^	0.91 (0.79; 0.96)^§^	0.10	0.14	−0.06 (−0.35; 0.23)	−0.05 (−0.47; 0.37)
	2nd_30 s	0.07 (−0.53; 0.67)*	0.10 (−0.50; 0.70)*	0.95 (0.89; 0.98)^§^	0.97 (0.92; 0.99)^§^	0.14	0.11	0.05 (−0.31; 0.40)	0.06 (−0.22; 0.34)
	2nd_60 s	0.12 (−0.48; 0.72)*	0.15 (−0.45; 0.75)*	0.95 (0.87; 0.98)^§^	0.95 (0.87; 0.99)^§^	0.14	0.14	0.08 (−0.30; 0.45)	0.09 (−0.25; 0.43)
2nd training camp (*n* = 20)	1st_30 s	0.24 (−0.28; 0.77)^#^	0.22 (−0.39; 0.84)^#^	0.86 (0.69; 0.94)^‡^	0.76 (0.52; 0.89)^‡^	0.24	0.31	0.16 (−0.42; 0.73)	0.20 (−0.56; 0.96)
	1st_60 s	0.16 (−0.36; 0.68)*	0.11 (−0.50; 0.73)*	0.91 (0.80; 0.96)^§^	0.82 (0.64; 0.91)^‡^	0.20	0.28	0.11 (−0.39; 0.61)	0.15 (−0.54; 0.85)
	2nd_30 s	0.06 (−0.46; 0.59)*	−0.07 (−0.68; 0.54)*	0.90 (0.79; 0.95)^§^	0.88 (0.76; 0.94)^‡^	0.20	0.22	0.04 (−0.51; 0.59)	0.09 (−0.49; 0.67)
	2nd_60 s	−0.03 (−0.49; 0.55)*	0.11 (−0.41; 0.63)*	0.92 (0.83; 0.96)^§^	0.91 (0.81; 0.96)^§^	0.18	0.19	0.02 (−0.47; 0.51)	0.07 (−0.44; 0.58)
3rd training camp (*n* = 17)	1st_30 s	0.22 (−0.34; 0.79)^#^	0.46 (−0.11; 1.04)^#^	0.93 (0.76; 0.97)^§^	0.84 (0.19; 0.95)^‡^	0.12	0.18	0.11 (−0.19; 0.41)	0.22 (−0.09; 0.52)
	1st_60 s	0.12 (−0.45; 0.68)*	0.33 (−0.24; 0.90)^#^	0.98 (0.94; 0.99)^§^	0.90 (0.52; 0.97)^§^	0.07	0.16	0.05 (−0.13; 0.24)	0.16 (−0.13; 0.45)
	2nd_30 s	−0.07 (−0.64; 0.49)*	0.11 (−0.45; 0.68)*	0.96 (0.92; 0.98)^§^	0.93 (0.84; 0.97)^§^	0.12	0.16	−0.04 (−0.34; 0.25)	0.06 (−0.33; 0.45)
	2nd_60 s	−0.07 (0.64; 0.49)*	0.11 (−0.45; 0.68)*	0.98 (0.94; 0.99)^§^	0.95 (0.88; 0.98)^§^	0.08	0.13	−0.04 (−0.27; 0.18)	0.06 (−0.25; 0.37)
4th training camp (*n* = 14)	1st_30 s	0.15 (−0.47; 0.77)*	0.17 (−0.45; 0.80)*	0.96 (0.87; 0.99)^§^	0.90 (0.77; 0.96)^§^	0.08	0.13	0.06 (−0.13; 0.26)	0.07 (−0.25; 0.40)
	1st_60 s	0.12 (−0.50; 0.74)*	0.14 (−0.48; 0.77)*	0.98 (0.93; 0.99) ^§^	0.94 (0.85; 0.98)^§^	0.06	0.10	0.05 (−0.08; 0.18)	0.06 (−0.20; 0.32)
	2nd_30 s	0.24 (−0.38; 0.87)^#^	0.27 (−0.35; 0.90)^#^	0.85 (0.64; 0.94)^‡^	0.90 (0.68; 0.96)^§^	0.16	0.13	0.10 (−0.31; 0.51)	0.11 (−0.18; 0.40)
	2nd_60 s	0.10 (−0.52; 0.72)*	0.12 (−0.50; 0.75)*	0.94 (0.86; 0.98) ^§^	0.97 (0.90; 0.97)^§^	0.10	0.07	0.04 (−0.22; 0.31)	0.05 (−0.10; 0.21)

The level of effect size was symbolled as trivial (0.0–0.2) as * and small (0.2–0.6) as ^#^. The level of interclass correlation coefficients was denoted small (0–0.3) as *, moderate (0.31–49) as ^#^, very large (0.70–89) as ^‡^, and nearly perfect (0.9–1) as ^§^. ES, effect size; ICC, interclass correlation coefficients; CI, confident interval; SEM, standard error of measurement; SD, standard deviation; n, numbers.

**Table 3:** Coefficient of variation of natural logarithm of root mean square differences between adjacent normal R–R intervals (LnRMSSD) during 0–30 s, 0–60 s, and 0–5 min criterion in stabilization (1st_5min) and after stabilization (2nd_5min) assessments.

**Training camp**	**Parameters**	**ES (90% CI)**		**ICC (90% CI)**		**SEM**		Bias (± 1.96_*_SD)	
		**1st_5 min**	**2nd_5 min**	**1st_5 min**	**2nd_5 min**	**1st_5 min**	**2nd_5 min**	**1st_5 min**	**2nd_5 min**
1st training camp (*n* = 15)	1st_30 s	−0.55 (−1.17; 0.05)^#^	−0.56 (−1.18; 0.05)^#^	0.38 (0.01; 0.68)^#^	0.23 (−0.16; 0.58)*	3.04	3.39	−2.14 (−10.07; 5.78)	−2.23 (−11.56; 7.10)
	1st_60 s	−0.34 (−0.95; 0.26)^#^	−0.35 (−0.97; 0.25)^#^	0.59 (0.23; 0.81)^†^	0.44 (0.37; 0.72)^#^	2.32	2.72	−1.28 (−7.67; 5.12)	−1.36 (−9.15; 6.42)
	2nd_30 s	−0.66 (−1.29; 0.05)^†^	−0.66 (−1.29; 0.06)^†^	0.40 (0.02; 0.69)^#^	0.44 (0.05; 0.72)^#^	4.80	4.63	−3.44 (−13.70; 6.83)	−3.52 (−13.38; 6.33)
	2nd_60 s	−0.56 (−1.18; 0.05)^#^	−0.57 (−1.19; 0.04)^#^	0.49 (0.10; 0.75) ^#^	0.53 (0.14; 0.78)^†^	3.98	3.82	−2.72 (−11.49; 6.05)	−2.81 (−11.16; 5.54)
2nd training camp (*n* = 20)	1st_30 s	−0.45 (−0.98; 0.08)^#^	−0.38 (0.90; 0.15)^#^	0.52 (0.20; 0.74)^†^	0.64 (0.35; 0.81)^†^	4.57	3.95	−2.85 (−14.35; 8.66)	−2.16 (−11.09; 6.78)
	1st_60 s	−0.13 (−0.65; 0.39)*	−0.02 (−0.54; 0.50)*	0.88 (0.76; 0.94)^‡^	0.81 (0.63; 0.91)^‡^	2.06	2.59	−0.80 (−6.44; 4.85)	−0.11 (−6.61; 6.40)
	2nd_30 s	−0.31 (−0.84; 0.21)^#^	−0.23 (−0.76; 0.29)^#^	0.76 (0.53; 0.88)^‡^	0.51 (0.18; 0.74)^†^	3.99	3.99	−2.24 (−11.30; 6.82)	−1.55 (−14.35; 11.25)
	2nd_60 s	−0.23 (−0.76; 0.29)^#^	−0.17 (−0.67; 0.38)*	0.82 (0.64; 0.91)^‡^	0.65 (0.37; 0.82)^†^	3.32	3.32	−1.63 (−9.43; 6.18)	−0.94 (−11.48; 9.60)
3rd training camp (*n* = 17)	1st_30 s	−0.65 (−1.24; 0.08)^†^	−0.87 (−1.48; 0.29)^†^	0.58 (0.13; 0.81)^†^	0.39 (−0.01; 0.68)^#^	3.32	4.01	−3.21 (−10.58; 4.16)	−4.06 (−12.71; 4.58)
	1st_60 s	−0.32 (−0.89; 0.25)^#^	−0.54 (−1.12; 0.03)^#^	0.81 (0.57; 0.92)^‡^	0.61 (0.23; 0.82)^†^	1.96	2.81	−1.45 (−6.28; 3.38)	−2.30 (−8.75; 4.15)
	2nd_30 s	−0.34 (−0.91; 0.23)^#^	−0.55 (−1.13; 0.02)^#^	0.69 (0.40; 0.85)^†^	0.63 (0.23; 0.83)^†^	2.68	2.93	−1.59 (−8.48; 5.31)	−2.44 (−8.86; 3.98)
	2nd_60 s	−0.11 (−0.68; 0.45)*	−0.31 (−0.88; 0.25)^#^	0.80 (0.59; 0.91)^‡^	0.83 (0.59; 0.93)^‡^	2.13	1.96	−0.53 (−6.34; 5.28)	−1.38 (−5.84; 3.07)
4th training camp (*n* = 14)	1st_30 s	−0.91 (−1.58; 0.27)^†^	−0.66 (−1.31; 0.03)^†^	0.51 (−0.03; 0.79)^†^	0.57 (0.11; 0.82)^†^	2.86	2.68	−3.33 (−8.59; 1.93)	−2.50 (−8.23; 3.22)
	1st_60 s	−0.54 (−1.18; 0.09)^#^	−0.31 (−0.94; 0.31)^#^	0.71 (0.23; 0.89)^‡^	0.77 (0.49; 0.90)^‡^	2.33	2.07	−2.05 (−6.51; 2.41)	−1.23 (−6.00; 3.55)
	2nd_30 s	−0.67 (−1.32; 0.04)^†^	−0.51 (−1.16; 0.11)^#^	0.23 (−0.14; 0.58)*	0.45 (0.06; 0.74)^†^	6.33	5.35	−3.79 (−16.82; 9.24)	−2.96 (−13.98; 8.06)
	2nd_60 s	−0.47 (−1.12; 0.15)^#^	−0.27 (−0.90; 0.35)^#^	0.51 (0.12; 0.77)^†^	0.76 (0.49; 0.90)^‡^	3.33	2.33	−1.95 (−9.31; 5.42)	−1.12 (−6.46; 4.22)

The level of effect size was symbolled as trivial (0.0–0.2) as *, small (0.2–0.6) as ^#^ and moderate (0.6–1.2) as ^†^. The level of interclass correlation coefficients was denoted small (0–0.3) as *, moderate (0.31–49) as ^#^, large (0.50–69) as ^†^ and very large (0.70–89) as ^‡^. ES, effect size; ICC, interclass correlation coefficients; CI, confident interval; SEM, standard error of measurement; SD, standard deviation; n, numbers.

**Figure 3:**
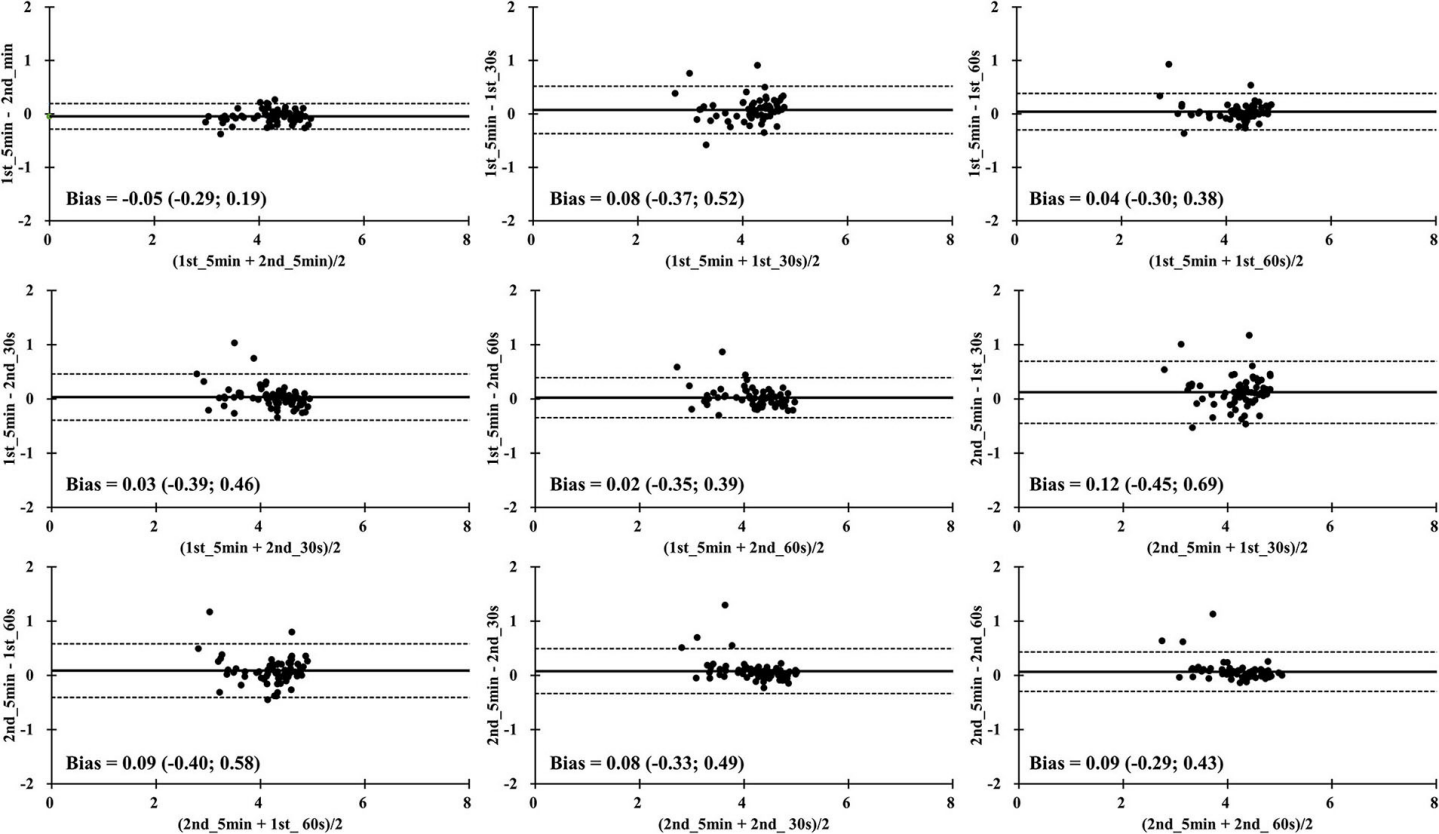
Bland-Altman plots for the mean of natural logarithm of the root mean square differences between adjacent normal R-R intervals in all time segments during four overseas training camps. 1st_5min = The first 5-min LnRMSSD; 1st_30 s = The first 30 s LnRMSSD; 1st_60 s = The first 60 s LnRMSSD; 2nd_5 min = The 6-10 min LnRMSSD; 2nd_30 s = The secondary 30 s LnRMSSD; 2nd_60 s = The secondary 60 s LnRMSSD.

**Figure 4:**
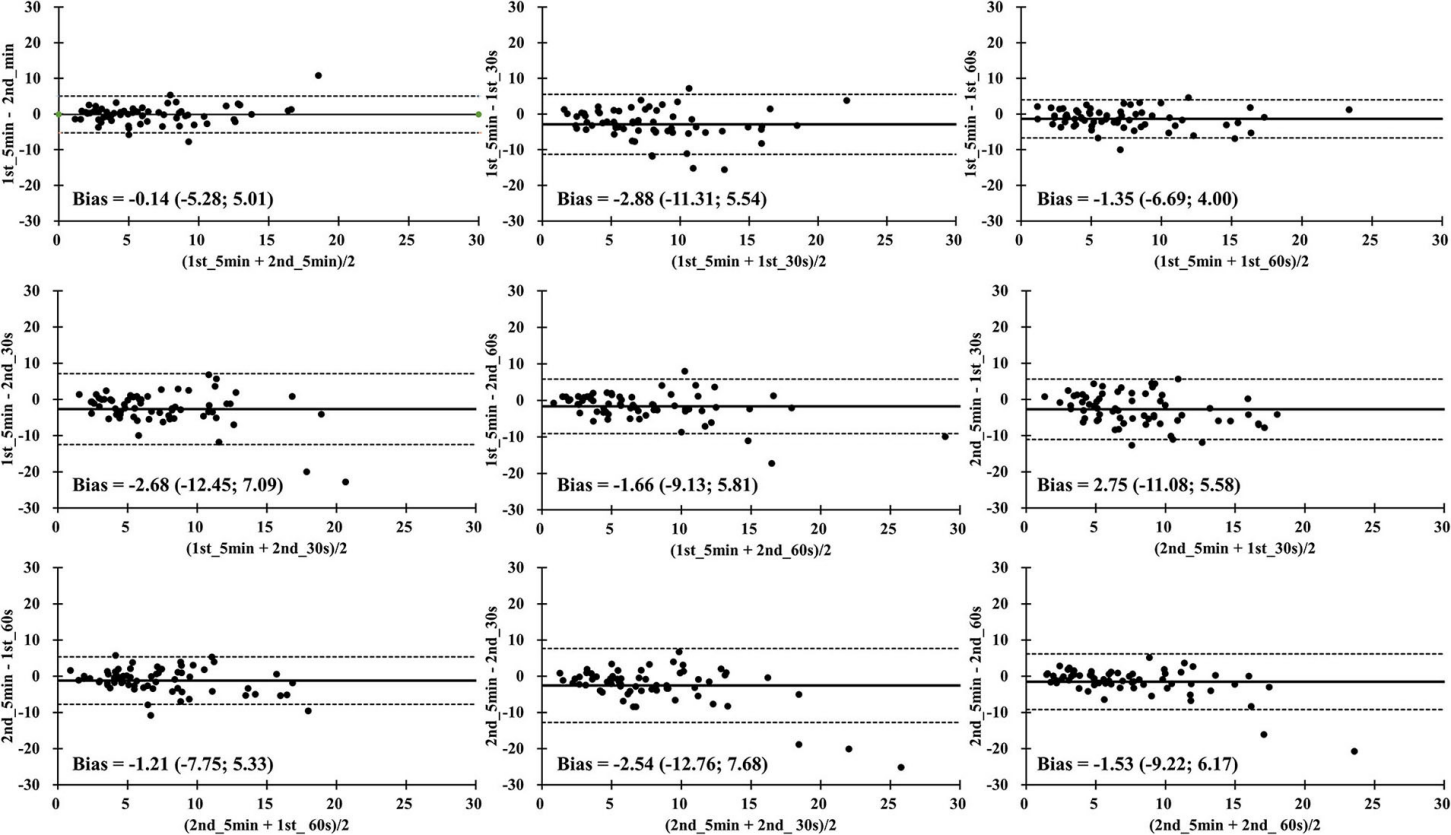
Bland-Altman plots for the coefficient of variation of natural logarithm of the root mean square differences between adjacent normal R-R intervals in all time segments during four overseas training camps. 1st_5 min = The first 5-min LnRMSSD; 1st_30 s = The first 30 s LnRMSSD; 1st_60 s = The first 60 s LnRMSSD; 2nd_5 min = The 6-10 min LnRMSSD; 2nd_30 s = The secondary 30 s LnRMSSD; 2nd_60 s = The secondary 60 s LnRMSSD.

In Figure 3, the results of LnRMSSD_mean_ show excellent limits of agreement in all time segment comparisons. The smallest mean difference and the upper and lower limits of agreements was found in the 1st_5 min and 2nd_5 min comparison (difference = −0.05, +1.96 s = −0.29, −1.96 s = 0.19). In Figure 4, the results of LnRMSSD_cv_ demonstrate a wide range of limits of agreement in all time segment comparisons.

### Comparison of LnRMSSD_mean_ and LnRMSSD_cv_ Among Overseas Training Camps

The descriptive results of LnRMSSD_mean_ and LnRMSSD_cv_ for all time segments during four different overseas training camps are presented in Table 4. Collectively, pairwise comparisons showed trivial to small ES in LnRMSSD_mean_ (0.02; 0.41 ES) and LnRMSSD_cv_ (−0.02; −0.49 ES) across overseas training camps.

**Table 4:** Natural logarithm of the root mean square differences between adjacent normal R–R intervals (mean and coefficient of variation values) in first 30 s, first 60 s, and 5 min time segments during stabilization vs after stabilization in four overseas training camps.

	**1st TC**	**2nd TC**	**3rd TC**	**4th TC**	**1 vs. 2 ES**	**1 vs. 3 ES**	**1 vs. 4 ES**	**2 vs. 3 ES**	**2 vs. 4 ES**	**3 vs. 4 ES**
**LnRMSSDmean**										
1st_5 min	4.21 ± 0.53	4.19 ± 0.57	4.22 ± 0.52	4.20 ± 0.40	0.04 (−0.53; 0.60)	−0.02 (−0.60; 0.56)	0.02 (−0.59; 0.63)	−0.05 (−0.60; 0.49)	−0.02 (−0.59; 0.55)	0.04 (−0.55; 0.64)
1st_30 s	4.27 ± 0.39	4.04 ± 0.64	4.11 ± 0.45	4.14 ± 0.40	0.41 (−0.15; 0.99)	0.37 (−0.21; 0.96)	0.32 (−0.29; 0.94)	−0.12 (−0.67; 0.42)	−0.18 (−0.75; 0.40)	−0.07 (−0.66; 0.52)
1st_60 s	4.28 ± 0.46	4.09 ± 0.67	4.16 ± 0.50	4.15 ± 0.42	0.32 (−0.25; 0.89)	−0.12 (−0.70; 0.47)	0.29 (−0.32; 0.91)	−0.11 (−0.66; 0.43)	−0.10 (−0.68; 0.47)	0.02 (−0.57; 0.62)
2nd_5 min	4.23 ± 0.54	4.24 ± 0.57	4.32 ± 0.45	4.21 ± 0.39	−0.02 (−0.58; 0.54)	−0.15 (−0.74; 0.43)	0.04 (−0.57; 0.65)	−0.15 (−0.70; 0.39)	0.06 (−0.51; 0.63)	0.25 (−0.34; 0.85)
2nd_30 s	4.17 ± 0.63	4.15 ± 0.65	4.26 ± 0.60	4.10 ± 0.41	0.03 (−0.53; 0.59)	−0.19 (−0.78; 0.39)	0.13 (−0.48; 0.74)	−0.17 (−0.72; 0.37)	0.09 (−0.49; 0.66)	0.30 (−0.29; 0.90)
2nd_60 s	4.14 ± 0.65	4.17 ± 0.63	4.26 ± 0.57	4.16 ± 0.40	−0.05 (−0.61; 0.52)	−0.19 (−0.78; 0.39)	−0.04 (−0.65; 0.58)	−0.15 (−0.69; 0.40)	0.02 (−0.56; 0.59)	0.19 (−0.40; 0.79)
**LnRMSSDcv**										
1st_5 min	7.01 ± 3.71	5.39 ± 5.91	7.35 ± 4.47	6.33 ± 2.96	0.31 (−0.25; 0.88)	−0.08 (−0.65; 0.50)	0.20 (−0.40; 0.80)	−0.36 (−0.92; 0.18)	−0.19 (−0.76; 0.39)	0.26 (−0.34; 0.86)
1st_30 s	9.15 ± 3.87	8.23 ± 6.59	10.56 ± 5.13	9.66 ± 4.09	0.16 (−0.40; 0.73)	−0.30 (−0.89; 0.28)	−0.13 (−0.74; 0.49)	−0.38 (−0.94; 0.16)	−0.25 (−0.82; 0.33)	0.18 (−0.41; 0.79)
1st_60 s	8.29 ± 3.63	6.18 ± 5.94	8.80 ± 4.49	8.38 ± 4.33	0.41 (−0.16; 0.98)	−0.12 (−0.71; 0.46)	−0.02 (−0.64; 0.59)	−0.49 (−1.07; 0.08)	−0.40 (−0.99; 0.17)	0.09 (−0.50; 0.69)
2nd_5 min	6.92 ± 3.92	6.08 ± 4.46	6.50 ± 3.89	7.15 ± 3.27	0.19 (−0.37; 0.76)	0.11 (−0.48; 0.69)	−0.06 (−0.67; 0.55)	−0.10 (−0.64; 0.44)	−0.26 (−0.84; 0.31)	−0.18 (−0.77; 0.42)
2nd_30 s	10.45 ± 6.19	7.63 ± 8.14	8.94 ± 4.81	10.11 ± 7.22	0.37 (−0.20; 0.96)	0.27 (−0.32; 0.87)	0.05 (−0.56; 0.66)	−0.19 (−0.74; 0.35)	−0.31 (−0.89; 0.26)	−0.19 (−0.79; 0.40)
2nd_60 s	9.73 ± 5.58	7.01 ± 7.84	7.88 ± 4.76	8.27 ± 4.75	0.38 (−0.19; 0.96)	0.35 (−0.18; 0.96)	0.27 (−0.34; 0.89)	−0.13 (−0.67; 0.41)	−0.18 (−0.76; 0.39)	−0.08 (−0.68; 0.51)

*Data is presented as mean and standard deviation or effect size and 90% confidence intervals. 1st_5 min = first 5–min LnRMSSD; 1st_30 s = first 30 s LnRMSSD; 1st_60 s = first 60 s LnRMSSD; 2nd_5 min = 6–10min LnRMSSD; 2nd_30 s = secondary 30 s LnRMSSD; 2nd_60 s = secondary 60 s LnRMSSD; TC, training camp; ES, effect size*.

## Discussion

This study is the first to report the reliability and validity of HRV_UST_ during a series of overseas training camps in male U-20 national team futsal players. The primary findings of the present study revealed that the 30 and 60 s HRV_UST_ measure during stabilization and post-stabilization periods were valid and acceptable measures for LnRMSSD assessment and can be used as a surrogate for the standard 5-min recording. In addition, the secondary finding in the present study showed trivial to small levels of effect size in all time segments of LnRMSSD_mean_ and LnRMSSD_cv_ across overseas training camps.

### Agreement and Reliability of Ultra-Short-Term Heart Rate Variability

The present study attempted to investigate the agreement of HRV_UST_ assessment for cardiac-autonomic adaptation during and after the stabilization process. The results of LnRMSSD_mean_ demonstrated trivial to small ES, very large to nearly perfect ICC, and narrow range of SEM (0.06–0.31) when all time segments of HRV_UST_ were compared to the 1st_5 min and 2nd_5 min HRV. Furthermore, the magnitude of the correlation coefficients was nearly perfect when the 1st_5 min was compared to all the time segments (*r* = 0.90–0.97). In terms of agreement of HRV_UST_, we found that there was excellent acceptance in all time segments. Indeed, perfect agreement was found between the 1st_5 min and 2nd_5 min HRV comparison (narrow risk of bias and limits of agreement). These findings indicate an absence of stabilization prior to HRV_UST_ measurement is acceptable for the accuracy of HRV measurement during overseas training camps. Our laboratory recently reported acceptance of 30-s HRV_UST_ of LnRMSSD_mean_ measure during short-term training camps in young adult futsal players (Chen et al., 2020). Nakamura et al. (2020) compared the limits of agreement between a standard 10 min LnRMSSD (5-min stabilization and 5-min HRV record) and LnRMSSD_UST_ (1–2 min record followed by 1-min stabilization) in 11 male futsal players before and after 4-weeks of pre-season training. Nakamura's et al. study showed meaningful changes in HRV_UST_ measures in response to a futsal pre-season (these results indicate a progressive increase in the vagal activity and reduction of training-induced perturbation of cardiac autonomic homeostasis over the futsal pre-season). Excellent agreement and reliability of HRV_UST_ after 1 min stabilization has also been reported in athletic populations (Esco and Flatt, 2014; Nakamura et al., 2015; Flatt and Esco, 2016; Pereira et al., 2016). Our observation further supports the notion of implementing HRV_UST_ recording by measuring weekly LnRMSSD_mean_ during overseas training camps due to the very high and nearly perfect correlation coefficient and a narrow range of limits of agreement during all time segments. Indeed, all players in our study were familiarized with the procedure of HRV measurement in order to increase the accuracy of recording. Collectively, our findings demonstrate consistent excellent agreement of HRV_UST_ of weekly LnRMSSD_mean_ during overseas training camps without the conventional 5-min postural stabilization period recommended when LnRMSSD is used for HRV recording.

The results of LnRMSSD_cv_ demonstrated trivial to large ES, small to very large ICC when HRV_UST_ parameters were used to compare the 1st_5 min and 2nd_5 min HRV. In addition, high to very high correlations and a wide range of limits of agreement in all time segments of LnRMSSD_cv_ comparisons were identified (Figures 2, 4). It seems that the shortened LnRMSSD_cv_ recording less than 1 min may increase the bias of measurement. Our laboratory recently reported that HRV_UST_ of LnRMSSD_cv_ could not be used as a surrogate of 5 min standard HRV records during short-term training camps due to inaccuracy of measures (Chen et al., 2020). It is important to note that LnRMSSD_cv_ increases in association with perception of fatigue and reduce of physical performance (Flatt et al., 2017a). Previous studies have demonstrated that 1-min LnRMSSD_cv_ measure after 1-min postural stabilization is sensitive to physical adaptation in response to periodization of training loads in women soccer players (Flatt and Esco, 2015), rugby seven players (Flatt and Howells, 2019), sprint swimmers (Flatt et al., 2017b), and futsal players (Nakamura et al., 2020). Interestingly, in our study, 60 s HRV_UST_ of LnRMSSD_cv_ demonstrated better ICC values, magnitude of correlation coefficient, and agreement of measures than 30 s HRV_UST_ of LnRMSSD_cv_ despite stabilization or after stabilization period. Nevertheless, cautions should be taken when LnRMSSD_cv_ are used to evaluate ANS adaptation during training camps.

### Comparisons of LnRMSSD_mean_ and LnRMSSD_cv_ Among Oversea Training Camps

As demonstrated in Table 4, in comparison with LnRMSSD_mean_ among overseas training camps, trivial ES was observed in 1st_5 min, 2nd_30 s, 2nd_60 s, and 2nd_5in time segments. In contrast, trivial to small ES in 1st_30 s and 1st_60 s time segments. Moreover, a wide range of ES between trivial and small levels were identified when LnRMSSD_cv_ variable were compared across overseas training camps. It is argued that LnRMSSD_mean_ should be used as a global marker to evaluate the training adaptation of vagal-related activities (Plews et al., 2013b). Whereas, LnRMSSD_cv_ is sensitive to detect the daily variation of ANS adaptation to training workloads and psychophysiological conditions (Nakamura et al., 2020). Our laboratory recently reported that HRV_UST_ of LnRMSSD_cv_ after stabilization period demonstrated large bias and invalid results. In contrast, HRV_UST_ of LnRMSSD_mean_ was valid and reliable to the 5-min standard measure (Chen et al., 2020). However, LnRMSSD_mean_ and LnRMSSD_cv_ were only examined in ultra-short-term and standard time segments with/without stabilization in the present study. Nevertheless, trivial to small ES found in all pairwise comparisons indicated the patterns of LnRMSSD_mean_ and LnRMSSD_cv_ modulation are similar despite the various objectives of overseas training camps.

### Limitations

The findings in the present study were limited by third factors. Firstly, the number of players vary from camps to camps. Longitudinal adaptation in vagal-related changes in relation to periodization was incomparable in this study Secondly, this study did not compare HRV responses to psychometric and physiological markers of training adaptation. Relationship between HRV_UST_ measures and psychophysiological responses during overseas training camps should be established in future studies. Thirdly, time difference and traveling stress within the fourth overseas training camp may have caused a potential bias in the interpretation of the outcome of the studies. Other logistical issues such as the traveling itinerary, accommodation, accessibility of sports facilities, local transportation, and kit management have a critical effect on players' recovery status.

### Practical Implication

It is understood that time management is a critical issue within training camps. The players require adequate rest time for recovery from the heavy burden of psychophysiological strains due to congested scheduling (i.e., training sessions, matches, meetings, dining, and additional team activities). These factors are considered when additional prerequisites such as medical checks, psychological consultations, rehabilitation, and training load monitoring are facilitated. The findings of this study suggest that HRV monitoring via HRV_UST_ measures within the first minute could be applied to assess vagal-related changes during training camp.

## Conclusion

In conclusion, either the first 30 or 60-s LnRMSSD recordings can be used to evaluate daily cardiac-autonomic functions during overseas training camps in futsal players. The process for stabilization seems to be unnecessary for measuring the morning resting LnRMSSD variable during overseas training camps in young adult futsal players. Consideration to use short HRV_UST_ measures should be addressed due to the discrepancies of LnRMSSD_cv_ between time segments. In addition, there is a trivial to small variation of weekly LnRMSSD_mean_ and LnRMSSD_cv_ across different overseas training camps. The observations in our study indicated specific characteristics of HRV modulation in U-20 futsal players during oversea training camps.

## Data Availability Statement

The raw data supporting the conclusions of this article will be made available by the authors, without undue reservation.

## Ethics Statement

The studies involving human participants were reviewed and approved by the Human Ethics Committee of the University of Taipei. Written informed consent to participate in this study was provided by the participants' legal guardian/next of kin.

## Author Contributions

Y-SC contributed to the study conceptualization, project administration, investigation, data analysis, methodology, and writing (including reviewing and editing) of the manuscript. JCP and PB contributed to the study conceptualization, data analysis, and writing (including reviewing and editing) of the manuscript. ZC-M contribute to the study data analysis and writing (including reviewing and editing) of the manuscript. C-DK and FMC contributed to the study conceptualization, methodology, supervision, and writing (including reviewing and editing) of the manuscript. All authors contributed to the article and approved the submitted version.

## Conflict of Interest

The authors declare that the research was conducted in the absence of any commercial or financial relationships that could be construed as a potential conflict of interest.
